# Discovery of the Cause, Nature, Cure and Prevention of Epidemic Cholera

**Published:** 1855-07

**Authors:** 


### DISCOVERY OF THE CAUSE, NATURE, CURE AND PREVENTION OF EPIDEMIC CHOLERA. By M. L. KNAPP, M. D.

THIS is a remarkable production. Asiatic cholera is "taken in and done for" with a dispatch that is worthy of note even in this fast age. We have seldom known anything so speedy. The cholera itself is hardly more rapid. Nay,

"With wings as swift
As meditation or the thoughts of love,"

Dr. Knapp has flown through the dark and impenetrable mystery which has hitherto enveloped the dread pestilence, and it now stands—to the eye of Dr. Knapp at least—before the world robbed of all its terrors, the most docile and harmless of entities. To have accomplished this, and that too with such amazing satisfaction to himself, as appears in Dr. Knapp's pamphlet, ought, in all reason, to be sufficient glory for any one mortal who was not a blood relation of the horse-leech. The contents of the production may be thus summarily stated: Cause, deficiency of vegetable food; nature, identical with scurvy; cure, esculents; prevention, good crops of potatoes, spinach, turnips, and other staple vegetables. If it would not be thought a work of supererogation we would, at the request of a German friend, respectfully suggest the cultivation on an extensive scale of *sour krout.*

